# Various Ocular Manifestations in Systemic Lupus Erythematosus: A Case Series

**DOI:** 10.7759/cureus.56503

**Published:** 2024-03-19

**Authors:** Muhammad Hazim Sharum, Rajasudha Sawri Rajan, Tajunisah Iqbal

**Affiliations:** 1 Ophthalmology, Hospital Selayang, Batu Caves, MYS; 2 Department of Ophthalmology, Faculty of Medicine, University of Malaya Eye Research Centre, Universiti Malaya, Kuala Lumpur, MYS

**Keywords:** systemic lupus erythematosus, choroidopathy, occlusive vasculitis, central retinal artery occlusion, central retinal vein occlusion

## Abstract

Systemic lupus erythematosus (SLE) is a chronic systemic autoimmune disorder with various systemic and ocular clinical manifestations. In patients with SLE, central retinal vein and artery occlusion, choroidopathy, and occlusive vasculitis are among the most significant and clinically relevant ocular manifestations, although they do not commonly occur.

We present a case series of three SLE patients of different races and genders who developed ocular-related clinical features of SLE during the course of their systemic disease. The clinical outcomes of each patient were different, affecting their vision in bilateral eyes, with some patients having better visual recovery while others having permanently poor vision. These outcomes were not significantly related to the patients’ age, gender, or race.

## Introduction

Autoantibody production and formation of circulating immune complexes are key features of systemic lupus erythematosus (SLE), a prevalent chronic autoimmune disorder that can result in organ dysfunction and tissue damage [1]. There are many manifestations of active SLE disease and these include the skin, serosal surfaces, kidneys, blood cells, central nervous system, and eyes. Immune complex deposition and cellular infiltration contribute to the development of vasculitis in SLE patients, leading to inflammation of and damage to retinal arterioles, capillaries, and venules [2]. As patients with evidence of retinal vasculitis reflect systemic vascular damage, close monitoring is important.

Severe vaso-occlusive retinopathy is a rare form of retinopathy that is often associated with poor visual prognosis [3]. Evidence indicates that patients afflicted with lupus and increased concentrations of antiphospholipid antibodies face an elevated likelihood of developing retinal vaso‐occlusive disease [4,5]. Such patients commonly present with profound bilateral visual loss.

Another less common ocular-related manifestation in SLE patients is choroidopathy with serous detachments of the retina, pigment epithelium, or both [6]. As SLE is a multisystem autoimmune disorder, it may have other ocular manifestations such as retinal hemorrhages (Putscher) and occlusive vasculitis. These changes may occur at the posterior pole or peripherally, without affecting visual acuity [7]. The estimated incidence of SLE ranges from 1.8 to 20 or more cases per 100,000 per year, and ocular manifestations commonly develop in patients who have active systemic disease [8].

Here, we present a case series of three SLE patients with multiple ocular clinical manifestations of both eyes during the course of their systemic disease.

This article was previously presented as a poster presentation at the 11th Conjoint Ophthalmology Scientific Conference Universiti Malaya-Asia Pacific Ophthalmic Trauma Society Conference, Kuala Lumpur on September 17th-18th, 2022.

## Case presentation

Case one

A 12-year-old Chinese girl with underlying SLE diagnosed in October 2021 initially presented in October 2021 with episodes of tiredness, loss of appetite, and mild loss of weight. Hematological investigations showed hemolytic anemia with hemoglobin of 5.3 g/dL, positive direct Coombs test, lactate dehydrogenase of 591 U/L, leucopenia with white blood cell count of 2.4 × 10^9^/L, and thrombocytopenia with platelet count 63 × 10^9^/L.

Serological results showed an antinuclear antibody of 1:1280 speckled, anti-dsDNA 681.4 IU/mL, positive anti-Ro, and raised erythrocyte sedimentation rate of 118 mm/hour. The Cardiolipin antibody test was negative. Lupus anticoagulant and activated partial thromboplastin time results were normal.

She presented to the ophthalmology clinic on November 8, 2021, complaining of sudden-onset painless blurring of vision in her right eye. On examination, her vision was counting fingers 1 feet OD and 6/24 OS. The relative afferent pupillary defect was positive over the right eye with a reduction in red saturation of 50% and light brightness of 20%.

**Figure 1:**
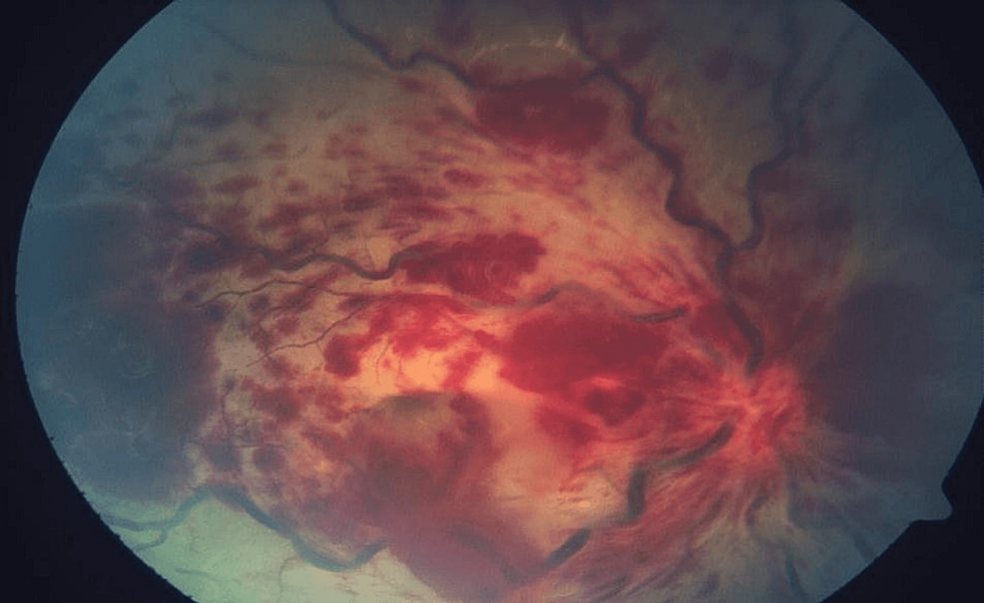
Fundus photograph of the right eye showing multiple flame-shaped hemorrhages with tortuous and dilated blood vessels. The pale swollen retina can be seen below.

Her right eye optic disc was swollen, and the retinal area showed multiple areas of retinal dot-blot hemorrhages, flame-shaped hemorrhage, pale retina, attenuated arteries, and venous tortuosity in all four quadrants (Figure 1). The left eye anterior segment and fundus were normal.

Fluorescein angiography of the right eye showed delayed filling of the central retinal artery, with complete retinal vein occlusion. She was diagnosed with right eye combined central retinal artery occlusion (CRAO) and central retinal vein occlusion (CRVO) secondary to SLE occlusive vasculitis. She was administered pulse intravenous methylprednisolone 500 mg once daily for three days followed by a tapering dose of intravenous (IV) methylprednisolone for two weeks. Subsequently, oral prednisolone 0.5 mg/kg was initiated. Laser pan-retinal photocoagulation was performed given the extensive area of capillary non-perfusion. Concurrently, she was started on IV cyclophosphamide with pediatric rheumatology.

**Figure 2:**
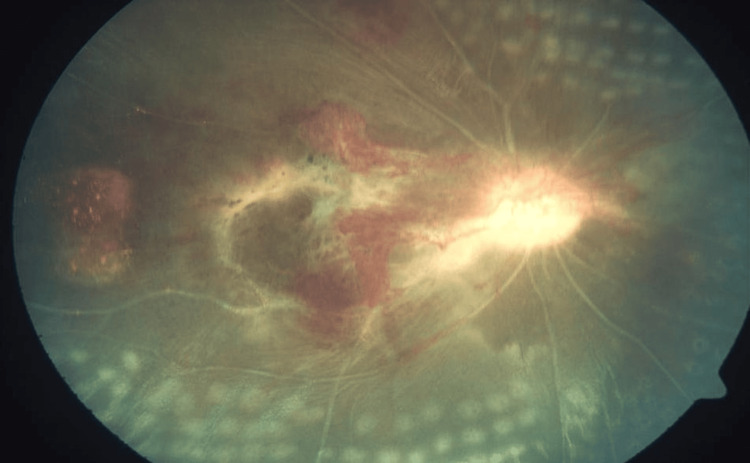
Fundus photograph of the right eye showing resolving hemorrhages at the posterior pole and sclerosed vessels with laser marks peripherally.

During follow-up, she reported progressive worsening of right eye vision. The latest assessment in February 2022 showed that her right eye vision had no perception to light (NPL). The fundus right eye revealed a pale optic disc, multiple flame-shaped hemorrhages at the posterior pole with ischemic retina, and sclerosed vessels in all quadrants with peripheral laser marks. There was no vitreous hemorrhage (Figure 2).

She was followed up by pediatric rheumatology and was still on schedule for cycles of IV cyclophosphamide administration. Despite systemic control with medications, the vision in her right eye remained poor with NPL OD.

Case two

A 15-year-old Malay girl with underlying SLE was admitted to the nephrology ward as she had active lupus nephritis with severe nephrosis and cerebral lupus with posterior reversible encephalopathy.

She presented with progressive bilateral blurring of vision for two weeks in February 2022. On examination, visual acuity in both eyes was 6/18. The anterior segments of both eyes were normal. Fundus examination in bilateral eyes showed a swollen optic disc, CDR 0.3, and there were multiple hypopigmented lesions at the posterior pole.

Retinal vessels were normal and no retinitis or vitritis was noted. Optical coherence tomography (OCT) of the macula in both eyes revealed an extensive amount of fluid in the subretinal spaces. On systemic review, she was noted to have uncontrolled hypertension with systolic blood pressure (BP) ranging from 136 mmHg to 170 mmHg and diastolic BP from 63 mmHg to 117 mmHg. She was diagnosed with bilateral eye SLE choroidopathy secondary to uncontrolled hypertension.

During ward admission, her BP was controlled with IV furosemide 80 mg TDS, followed by oral furosemide 80 mg TDS tapering dose, tab bisoprolol 10 mg OD, and tab amlodipine 10 mg OD. Before discharge from the nephrology ward, her systolic BP ranged from 127 mmHg to 130 mmHg and diastolic BP from 85 mmHg to 93 mmHg.

**Figure 3:**
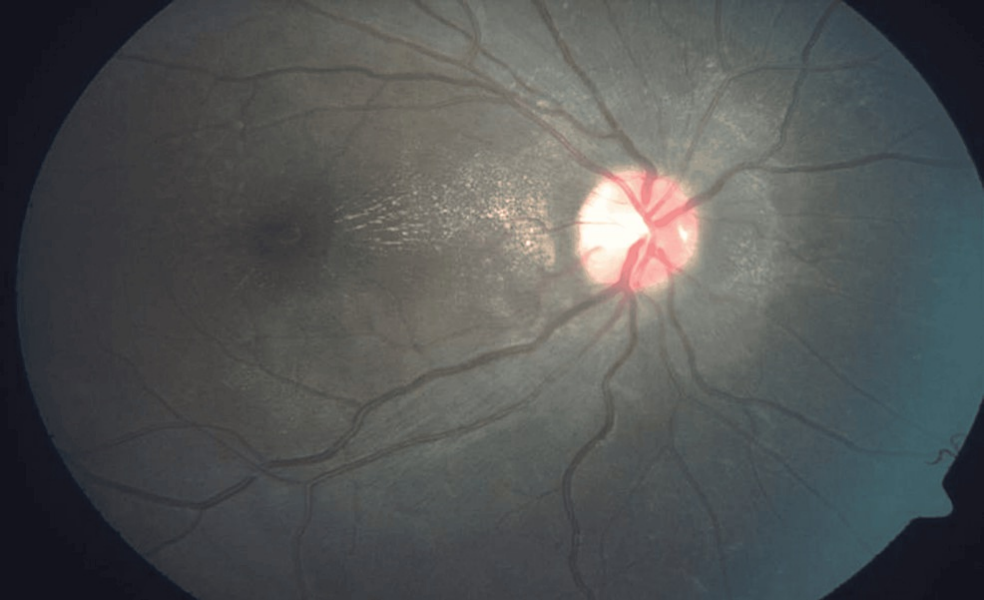
Fundus photograph of the right eye showing blurred disc margin and dull foveal light reflex with a macular star.

Upon review in the ophthalmology clinic at one month, her vision had improved to 6/12. Bilateral OD swelling had reduced with new findings of macular star in the right eye (Figure 3). The hypopigmented lesions were resolving. OCT of the macula of bilateral eyes showed a significant reduction in subretinal fluid.

Case three

A 12-year-old Malay boy with underlying SLE with secondary cerebral lupus presented with bilateral eye blurring of vision for one month. Visual acuity in both eyes was 6/36.

**Figure 4:**
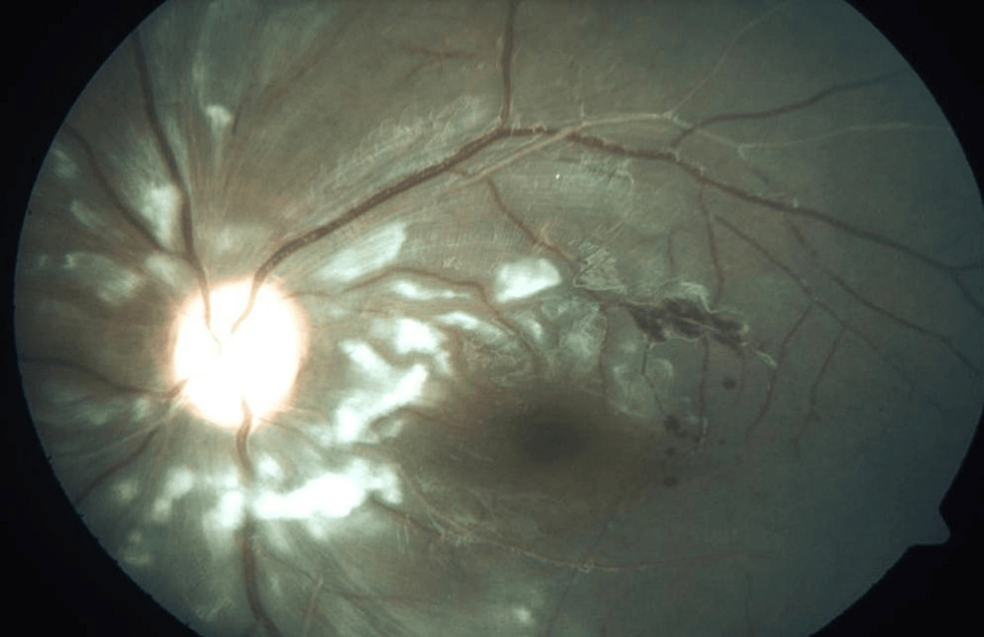
Left eye fundus photograph showing multiple intraretinal hemorrhages at the macula with macula edema.

The right eye fundus showed a pale OD with CDR 0.3, edematous macula, multiple cotton wool spots, and attenuation of retinal arteries. Fundus examination of the left eye showed a pink optic disc with CDR 0.3, edematous macula, multiple cotton wool spots, and deep retinal hemorrhages (Figure 4). Extensive retinal artery attenuation was also seen. No retinal neovascularization was noted.

**Figure 5:**
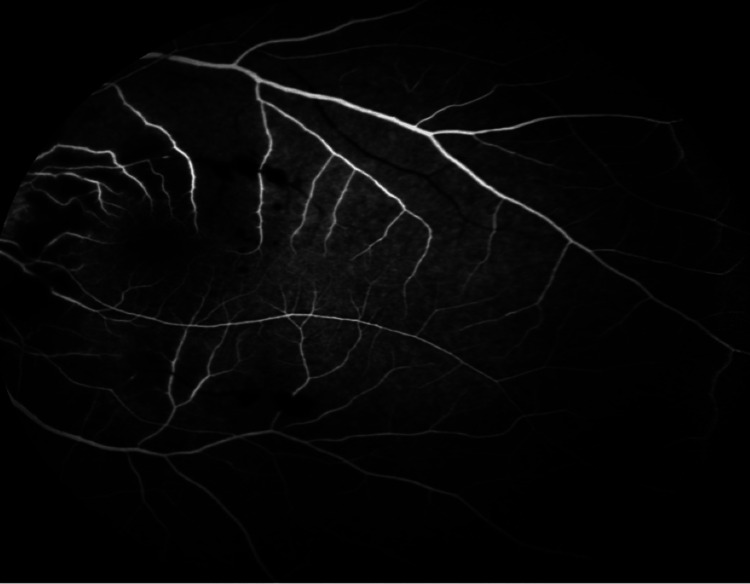
Fundal fluorescein angiography of the left eye showing a hypofluorescence area at the macula owing to intraretinal hemorrhages with poor arterial and venous filling.

Fluorescein angiography showed delayed and poor arterial and venous filling (Figure 5).

There were large areas of capillary non-perfusion in both eyes with involvement of the macula in the right eye. He was diagnosed with bilateral eye occlusive vasculitis involving artery and veins with right eye CRAO. A bilateral pan-retinal photocoagulation (PRP) laser was initiated until completion.

The latest examination showed improvement in best-corrected vision to 6/24 OD and 6/9 OS. The right eye fundus showed a pale optic disc with CDR 0.7, a venous loop at the superotemporal arcade, and peripheral PRP laser marks.

The left eye optic disc was pale with CDR 0.5 and PRP laser marks at the periphery. No new vessels were seen at the optic disc or elsewhere in both eyes. There were no features of vitritis, vasculitis, or retinitis. No macula edema was noted in either eye. He is also being followed up by pediatric rheumatology and is currently on tab prednisolone 5 mg OD and tab azathioprine 100 mg OD.

## Discussion

This case series presents the association between SLE patients and their elevated risk of developing ocular complications. Patients with SLE are frequently seen to have retinal vascular changes with retinal hemorrhage and reduced vision [8]. Vaso-occlusive retinopathy is rarely seen; however, it may occur and is often associated with antiphospholipid syndrome and neovascularization with poor visual prognosis [9]. Studies have demonstrated that individuals diagnosed with lupus and elevated levels of antiphospholipid antibodies are more susceptible to the development of retinal vaso‐occlusive disease [10].

The first patient in our case series developed combined CRAO and CRVO in her right eye with poor visual outcome. Occlusions of central retinal artery (CRAO) or vein (CRVO) and combined forms secondary to SLE have been reported in the literature and are usually unilateral [10].

Combined CRAO and CRVO have been reported in a variety of clinical settings, including in association with syphilis [11], Behcet’s disease [12], cat-scratch disease [13], orbital inflammatory pseudotumor [14], and posterior scleritis [15].

Ischemia in the retina, caused by a simultaneous blockage of both the central retinal artery and central retinal vein in the right eye, led to a devastating loss of vision and the proliferation of new blood vessels. Such a clinical manifestation in this case demonstrates that lupus retinopathy is a potential cause of visual morbidity.

Another serious form of ocular complication in SLE patients is choroidopathy with serous detachment of the retina, pigment epithelium, or both. This is uncommon and is usually seen in uncontrolled hypertension patients. Immune complex deposition in the choriocapillaris and the presence of autoantibody directed against retinal pigment epithelium with subsequent fluid leakage into the subretinal space are thought to play a role in the pathogenesis of lupus choroidopathy [16,17].

Fluorescein angiography shows delayed choroidal filling owing to the deposition of antigen-antibody immune complexes in the vessel walls [16,18].

The patient in our second case had lupus nephritis with uncontrolled hypertension, leading to lupus choroidopathy in both eyes. Clinical examination and investigation with OCT of the macula also revealed serous retinal detachment and choroidal involvement. It was clear that uncontrolled hypertension induced by the patient’s lupus nephritis contributed to the retinopathy and choroidopathy.

The confounding factor of hypertensive retinopathy aside, we attribute the occurrence of retinopathy and choroidopathy in this patient to her underlying etiology of active SLE, leading to hospitalization, and improving when the blood pressure was under control with antihypertensive medication and lupus disease activity suppressed with aggressive corticosteroids. Inflammation certainly plays a major role in the development of choroidopathy in lupus [19].

Hypertension damages the choroidal vessels and contributes to serous retinal detachments. Therefore, choroidopathy in lupus results from a combination of processes such as inflammation, thrombosis, and hypertension. Our second case suggests that, when lupus is active with increased severity of nephropathy, clinicians should be concerned about the risk of choroidal involvement.

Retinal occlusive vasculitis is another commonly associated ocular complication in SLE patients. The presence of retinal vasculitis with coexisting cerebral lupus is a feature of more active disease [16].

Our third patient presented a rare case that highlights the signs of retinopathy and vasculopathy. The patient showed bilateral eye occlusive vasculitis involving artery and veins with right eye CRAO.

Immune complex deposition in the vessel wall leads to the development of retinal occlusive vasculitis in SLE, which can be confirmed through immunofluorescent techniques, showcasing the accumulation of immune complexes at choroidal capillaries during autopsy [16].

The adhesion of immunocomplex molecules involving small vessels leads to vascular inflammation [19] and can be a compounding factor in this case, explaining the involvement of the peripheral retinal vessels.

In our case, there was development of cerebral lupus with retinal manifestations of SLE, thus suggesting active relapse of the underlying vascular inflammation. Fluorescein angiography is significant in determining the degree of active vasculopathy. Active vasculitis caused the area of macula ischemia seen in angiography in the patient’s right eye, necessitating treatment with laser photocoagulation. Our patient in this case was treated with an intravenous immunosuppressive agent as well as with oral steroids for good disease control.

## Conclusions

A comprehensive analysis of the ocular symptoms manifested by SLE patients, documented in all the aforementioned cases, enhances our understanding of the potential complications that can manifest in uncontrolled lupus. By utilizing this information, prompt and efficient medical intervention can be performed to avoid any complications that could potentially affect the patient’s vision, while also coordinating the required examinations and systemic treatment involving immunosuppressive agents. To achieve optimal outcomes, ophthalmologists and rheumatologists play a major role in assessing the disease activity of SLE patients to deliver effective management.

## References

[1] Kotzin BL (1996). Systemic lupus erythematosus. Cell.

[2] Mills JA (1994). Systemic lupus erythematosus. N Engl J Med.

[3] Mendrinos E, Mavrakanas N, Kiel R, Pournaras CJ (2009). Bilateral combined central retinal artery and vein occlusion in systemic lupus erythematosus resulting in complete blindness. Eye (Lond).

[4] Ushiyama O, Ushiyama K, Koarada S (2000). Retinal disease in patients with systemic lupus erythematosus. Ann Rheum Dis.

[5] Asherson RA, Merry P, Acheson JF, Harris EN, Hughes GR (1989). Antiphospholipid antibodies: a risk factor for occlusive ocular vascular disease in systemic lupus erythematosus and the 'primary' antiphospholipid syndrome. Ann Rheum Dis.

[6] Jabs DA, Hanneken AM, Schachat AP, Fine SL (1988). Choroidopathy in systemic lupus erythematosus. Arch Ophthalmol.

[7] Md Noh UK, Zahidin AZ, Yong TK (2012). Retinal vasculitis in systemic lupus erythematosus: an indication of active disease. Clin Pract.

[8] Arevalo JF, Lowder CY, Muci-Mendoza R (2002). Ocular manifestations of systemic lupus erythematosus. Curr Opin Ophthalmol.

[9] Au A, O'Day J (2004). Review of severe vaso-occlusive retinopathy in systemic lupus erythematosus and the antiphospholipid syndrome: associations, visual outcomes, complications and treatment. Clin Exp Ophthalmol.

[10] Durukan AH, Akar Y, Bayraktar MZ, Dinc A, Sahin OF (2005). Combined retinal artery and vein occlusion in a patient with systemic lupus erythematosus and antiphospholipid syndrome. Can J Ophthalmol.

[11] Smith JL (1973). Acute blindness in early syphilis. Arch Ophthalmol.

[12] Richards RD (1979). Simulataneous occlusion of the central retinal artery and vein. Trans Am Ophthalmol Soc.

[13] Gray AV, Michels KS, Lauer AK, Samples JR (2004). Bartonella henselae infection associated with neuroretinitis, central retinal artery and vein occlusion, neovascular glaucoma, and severe vision loss. Am J Ophthalmol.

[14] Foroozan R (2004). Combined central retinal artery and vein occlusion from orbital inflammatory pseudotumour. Clin Exp Ophthalmol.

[15] Shukla D, Mohan KC, Rao N, Kim R, Namperumalsamy P, Cunningham ET Jr (2004). Posterior scleritis causing combined central retinal artery and vein occlusion. Retina.

[16] Nguyen QD, Uy HS, Akpek EK, Harper SL, Zacks DN, Foster CS (2000). Choroidopathy of systemic lupus erythematosus. Lupus.

[17] Cunningham ET Jr, Alfred PR, Irvine AR (1996). Central serous chorioretinopathy in patients with systemic lupus erythematosus. Ophthalmology.

[18] Schwartz MM, Roberts JL (1983). Membranous and vascular choroidopathy: two patterns of immune deposits in systemic lupus erythematosus. Clin Immunol Immunopathol.

[19] Cid MC (2002). Endothelial cell biology, perivascular inflammation, and vasculitis. Cleve Clin J Med.

